# A reflection on economic uncertainty and fertility in Europe: The Narrative Framework

**DOI:** 10.1186/s41118-020-00094-3

**Published:** 2020-09-09

**Authors:** Daniele Vignoli, Raffaele Guetto, Giacomo Bazzani, Elena Pirani, Alessandra Minello

**Affiliations:** Department of Statistics, Computer Science, Applications “G. Parenti” (DiSIA), University of Florence, Viale Morgagni, 59, 50134 Florence, Italy

## Abstract

The generalized and relatively homogeneous fertility decline across European countries in the aftermath of the Great Recession poses serious challenges to our knowledge of contemporary low fertility patterns. In this paper, we argue that fertility decisions are not a mere “statistical shadow of the past”, and advance the Narrative Framework, a new approach to the relationship between economic uncertainty and fertility. This framework proffers that individuals act *according to* or *despite *uncertainty based on their “narrative of the future” – imagined futures embedded in social elements and their interactions. We also posit that personal narratives of the future are shaped by the “shared narratives” produced by socialization agents, including parents and peers, as well as by the narratives produced by the media and other powerful opinion formers. Finally, within this framework, we propose several empirical strategies, from both a qualitative and a quantitative perspective, including an experimental approach, for assessing the role of narratives of the future in fertility decisions.

## Introduction

**Fig. 1:**
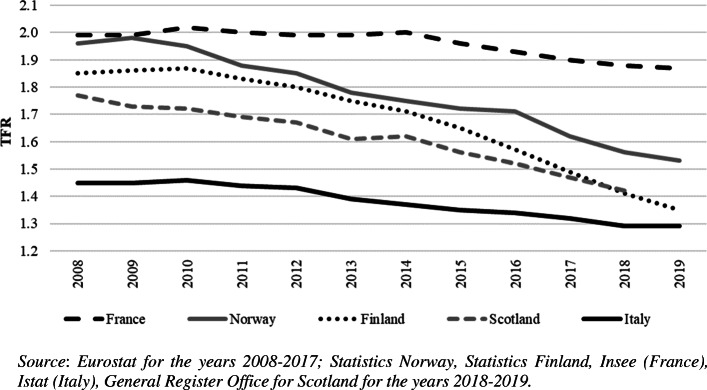
A few examples of the contemporary fertility winter in Europe. Total Fertility Rate (TFR) for Finland, France, Italy, Norway, and Scotland (2008-2019)

Contemporary Europe is facing a new fertility winter. In 2010, the already low fertility rates of Southern European countries started to decline again, and in recent years Nordic countries, too, have experienced a dramatic decrease in total fertility (Fig. 1). From a peak of almost two children per woman in 2009, in Norway the Total Fertility Rate (TFR) fell to 1.53 in 2019, the lowest in its history; Finland is facing a similar negative trend, with the record-low level of 1.35 in 2019. Finland is thus, perhaps surprisingly, approaching the “lowest-low fertility regime” (Kohler, Billari, Ortega 2002). Scotland, representative of the countries with medium fertility levels, is experiencing a slow but continuous fertility decline, losing approximately 0.30 TFR points in the last decade. France is the only European country with a TFR above 1.8, but there, too, the trend is unexpectedly downwards. In the South of Europe, Italy – and a similar trend is to be found in Spain and Greece – after a fertility rebound at the beginning of the new millennium, is now experiencing a constant fertility decline, officially re-entering the lowest-low regime in 2019, with a TFR of 1.29. This is closer to the negative record of 1995 (1.19) than to the fertility rebound of 2008 (1.45). Whereas some years ago the literature (prudently) forecast a slight rebound in completed fertility in Northern and Western Europe (Schmertmann et al. 2014), more recent research suggests instead that, at least for Finland, the all-time low period fertility currently observed is not a consequence of accelerating fertility postponement. Rather it is most likely a sign of a decreasing fertility *quantum* (Hellstrand, Nisén, and Myrskylä 2020).

This relatively homogenous decline in fertility across European countries is surprising because major perspectives in family demography – and most notably the Gender Revolution (Goldscheider, Bernhardt, and Lappegård 2015)/Multiple Equilibrium framework (Esping-Andersen and Billari 2015) – suggest, rather, a fertility rebound. In this reflection, we dispute that the central explanation for this new state of affairs is the rise of economic uncertainty. Fertility decisions are always taken in a condition of *fundamental uncertainty*, a condition in which the effects of present actions cannot be forecast or estimated with any confidence (Beckert 2016; Beckert and Bronk 2018). However, the increasing speed, dynamics, and volatility of outcomes from globalization, and new waves of technological change, makes it increasingly difficult for individuals to imagine their future, choose between alternatives, and form strategies (Mills and Blossfeld 2013). This generates a potent additional source of *economic uncertainty,* which, we argue, represents a *game-changer* in contemporary fertility dynamics.

The empirical demographic tradition operationalized the forces of economic uncertainty through objective indicators of individuals’ labor market situation, such as holding a temporary contract or being unemployed (Kreyenfeld 2010; Kreyenfeld, Andersson, and Pailhé 2012; Vignoli, Drefahl, and De Santis 2012; Mills and Blossfeld 2013; Kreyenfeld 2015; Busetta, Mendola, and Vignoli 2019; Vignoli, Tocchioni, and Mattei 2019). Nonetheless, although certainly worth taking into account, their (negative) impact on fertility has been shown not to be of overwhelming importance (Alderotti et al. 2019). A major shortcoming in such operationalizations of economic uncertainty is their “*backwards reasoning*” (Johnson-Hanks et al. 2011: 58): indicators and statistical models take fertility as the result of what already happened in the life course. But fertility decisions, we argue, are so much more than a “*statistical shadow of the past*” (Davidson 2010: 17; Beckert and Bronk 2018).

Recent advances also consider subjective measures of employment uncertainty (Kreyenfeld 2010; Bhaumik and Nugent 2011; Hofmann and Hohmeyer 2013; Fahlén and Oláh 2018), and individuals’ idiosyncratic preferences and psychological characteristics such as generalized trust, subjective well-being, risk aversion, and time-discounting preferences (see, e.g., Schmidt 2008, Gatta et al. 2019; Vignoli, Mencarini, and Alderotti 2020). However, beside the subjective perception of individuals’ security over their actual employment and economic situation, actors are influenced in their choices by more or less uncertain expectations about the consequences of a given action, a type of uncertainty that we might call the “*shadow of the future*” (Bernardi, Huinink, and Settersten 2019: 4).

In this article, we offer a trans-disciplinary contribution by extending the sociological work of Beckert (2016) on decision-making in conditions of uncertainty to fertility research. We argue that economic uncertainty needs to be conceptualized and operationalized taking into account that people use works of imagination, producing their own *narrative of the future* – namely, imagined futures embedded in social elements (individuals, organizations, and so forth) and their interactions. These personal narratives of the future are anchored in existing cultural and institutional frames, as well as in public images produced by the media and by other powerful opinion formers. Based on socially-constructed perceptions, people build their narratives of the future so as to take decisions in a condition of uncertainty. Narratives of the future allow people to act *according to* or *despite* the uncertainty they face, irrespective of structural constraints and their subjective perceptions.

The present paper aims to advance narratives of the future as a decisive lens in understanding the link between economic uncertainty and fertility in contemporary Europe. To this end, the study: (i) presents the theoretical relations between narratives, uncertainty and fertility; (ii) introduces the role of parental and media narratives as two major building blocks of the personal narratives of the future; and iii) offers empirical directions for future research. We concentrate on fertility intentions, but our approach can also be usefully applied to fertility behavior. Fertility intentions follow the desire for childbearing and anticipate concrete behavior by reflecting the combined effect of desired fertility and situational constraints (Thomson and Brandreth 1995; Billari, Philipov, and Testa 2009). Questions about intentions that cover a foreseeable time period (Ajzen & Fishbein, 1973) are generally considered to be good predictors of behavior (Westoff and Ryder 1977; Rindfuss, Morgan, and Swicegood 1988; Schoen et al. 1999; Billari et al. 2009; Régnier-Loilier and Vignoli 2011).

In the following sections, we first highlight open questions in current fertility research and review prior evidence on the economic uncertainty–fertility nexus. Then, we present our theoretical framework – i.e. the Narrative Framework – in relation to other prominent theoretical models of fertility and, focusing on the importance of *shared narratives*, we describe the building blocks of personal narratives. Afterwards, we offer a toolkit for the qualitative, quantitative and experimental analysis of personal narratives in fertility research. We conclude by discussing how the Narrative Framework may help in understanding the fertility decision-making process in an era of global uncertainty.

## Economic uncertainty as a game-changer for European fertility dynamics

### Puzzling European fertility patterns and trends

Important questions about the causes of current fertility declines remain unanswered and pose serious challenges to our knowledge on patterns of low, very low, and lowest-low fertility in Europe. Beyond structural economic indicators, labor market regulations, welfare regimes, educational systems and gender equality arguments have all been widely used as the key explanatory factors in fertility trends (see Balbo, Billari, and Mills 2013 for a review). While these explanations may account for several cross-country differences in fertility rates, the diffusion of economic uncertainty need to be integrated into current demographic approaches. Let us discuss the UK, and Nordic and Southern European countries as examples of different kinds of reactions to rising economic uncertainty.

The UK has long been seen as the clearest example of a European liberal market economy (Esping-Andersen 1999; Thévenon 2011). The forces of globalization would be expected to especially affect the economy of a country of this type with early and large-scale labor market deregulation and, compared to other European countries, a *light* welfare system. UK citizens have a high level of labor market instability compared to Central (e.g., France, Germany, and Austria) or Southern (e.g., Italy, Spain, and Greece) European countries. During the 1980 and 1990s, in West Germany, for instance, youth labor market entrance was found to be more direct and stable than in the UK, thanks to the high job protection and features of the educational system. In the UK, on the other hand, early-career occupational positions were more transitory and the quality of the job-match lower (Scherer 2005). This uncertainty was not, however, followed by a considerable gap in Britain’s TFR, which remained stable at about 1.8 children per woman. On the contrary, recent flexibilization reforms carried out both in the German and in Southern European labor markets have been linked to a strong postponement of first childbirth (Schmitt 2012; Barbieri et al. 2016). If employment uncertainty represents a crucial force influencing fertility decisions, why is its effect not visible in the UK’s fertility trend over the last decades?

As mentioned above, after the onset of the Great Recession – i.e. the 2007-2009 global financial, economic, and labor market decline – the TFR decreased in most EU countries. In Southern countries, people reasonably felt a high level of uncertainty in terms of opportunities due to fewer job prospects and less state protection (Matysiak, Sobotka, and Vignoli 2020). These structural forces did not shape up in the same way in Nordic societies. Apart from Iceland, Nordic countries did not experience an economic recession or crisis to the same extent as the rest of Europe: their Gross Domestic Product (GDP) has increased year in year out. However, their TFR decreased after 2009-2010, in a similar fashion to Southern European countries. Norway has switched, in fact, from being one of the countries with the highest fertility levels in Europe to having only average fertility levels. The observed decline in fertility has been unexpected and sharp, and similar changes have been observed in Sweden, Finland, and Denmark (Comolli et al. 2019). What are the driving forces for fertility decline in Nordic countries after a Great Recession that, in “hard numbers”, they barely experienced?

Recent trends in TFRs in Europe pose serious challenges to demographic knowledge because contemporary theories failed to foresee the fertility crisis. In line with McDonald (2000) and Goldscheider, Bernhardt, and Lappegård (2015), Esping-Andersen and Billari (2015) recently suggested that trends toward (lowest-)low fertility would be transitory, and that fertility rates would come back to levels close to the replacement threshold especially in countries where gender equality gains normative status. After all, Nordic countries represent a benchmark in terms of gender equality and female labor market participation (Guetto, Luijkx, and Scherer 2015), thus, the strong decrease in fertility in these countries is particularly unexpected within their Multiple Equilibrium framework. In an updated version of his thesis, Lesthaeghe (2010) recognized that some of the cultural components of the Second Demographic Transition (SDT) – such as self-actualization and consumption/leisure aspirations – cause a postponement in fertility. But he also pointed out that other components – such as the emancipation of young adults and gender symmetry in daily life – should mean a recuperation of the same. Again, Scandinavian countries are recognized for their promotion of youth emancipation (Billari 2004) and a higher prevalence of SDT-related forms of family behavior (Lesthaeghe 2020), factors that have been associated to higher fertility rates (Prskawetz, Mamolo, and Engelhardt 2010). Another surprising development has been the return to lowest-low fertility rates in Southern Europe. Castiglioni and Dalla Zuanna (2009) showed that Italy was approaching other Western European countries in terms of the diffusion of cohabitation and divorce, arguing that this was contributing to higher fertility rates. Billari (2008) related the shift toward a partly unexpected SDT to higher fertility rates in the case of Spain, too. All in all, recent fertility trends represent a challenge for Southern Europe as well (Comolli 2017; Caltabiano et al. 2019), as countries like Spain and Italy were deemed to have bounced back for good from lowest-low fertility rates (Billari and Dalla Zuanna 2008).

### The rise of economic uncertainty: Objective or perceived changes?

The impact of economic uncertainty on demographic behavior has been a centerpiece of the social sciences for over a century. However, a “*harsh new world of economic insecurity*” (Hacker 2019: xvi) only appeared in the 1980s, due to an array of global transformations, often included under the umbrella of “globalization”. This included: the declining importance of national borders for economic transactions; the intensification of worldwide social relations through the information and technology revolution; tougher tax competition between countries accompanied by the deregulation, privatization, and liberalization of domestic industries and markets; not to mention the rising importance of exposure to a volatile labor market (Jameson and Miyoshi 1998; Guillen 2001; Raab et al. 2008; Barbieri and Bozzon 2016). The promises of globalization – such as more competitive prices, wider choice, greater freedom, higher living standards and greater prosperity – had also other consequences, however. The best evidence suggests that individuals have become increasingly vulnerable to economic uncertainty, often being trapped into more precarious and lower-quality forms of employment such as fixed-term contracts and involuntary part-time work, or lower occupational standards (Blossfeld and Hofmeister 2006; Blossfeld, Mills and Bernardi 2006; Barbieri et al. 2016). The young especially are often viewed as the *losers of globalization* (Mills and Blossfeld 2013) and the emerging *precariat class* (Standing 2012). The volatile markets and the recent Great Recession have fueled the view that globalization is unpredictable (Grusky et al. 2011) and *out of control* (Mills and Blossfeld 2013). These kinds of uncertainty are expected to affect family formation, and are now viewed as drivers behind the postponement of childbearing and the elimination of higher-order parities in contemporary Europe (Kreyenfeld et al. 2012).

Beyond increasing instability in individuals’ work lives, globalization, and the neo-liberal policies that accompanied it, have also seen an increase in income inequality (OECD 2011). Income inequality has been on the rise since the late 1970s in most Western countries. While they are not the same thing, inequality and uncertainty are strongly interwoven: as rising income inequality may mean reduced intergenerational upward mobility (Barone 2019; Hacker 2019). Compared to previous generations, the young dealing with globalized labor markets, especially those from lower socioeconomic backgrounds, are less likely to improve on the level of socioeconomic well-being reached by their parents. According to Easterlin’s hypothesis on the role of *relative affluence* for fertility decisions (1976) – see also “Building-blocks of personal narratives: intergenerational and peers’ narratives” sub-section – this state of affairs may hinder family plans.

Empirical studies that simultaneously include several indicators of economic conjuncture – such as the unemployment rate, the economic policy uncertainty index, the cost of public debt, and the consumer confidence index – do not explain all the decline in birth rates in Europe and the US in the period 2008-2013 (e.g., Goldstein et al. 2013; Comolli 2017). A recent study by Matysiak, Sobotka and Vignoli (2020), illustrates that the negative impacts of economic conditions on fertility were more pronounced during the recession, than before. This intensification of the influence of economic conditions on fertility during the recession, however, did not result in the fact that Europeans adjust their fertility more strongly to worsening economic conditions rather than to the improvement of the same.

Clearly, there is something that is not captured by traditional economic and labor market indicators and that drives contemporary European fertility trends. Part of the unexplained fertility decline in the aftermath of the Great Recession can be explained by the rise of *perceived* economic uncertainty. Economic constraints directly affect many families and individuals by dragging down their income, but also by fueling general perceptions of uncertainty about future economic conditions (Kreyenfeld 2010; Vignoli, Rinesi and Mussino 2013; Raymo and Shibata 2017), even among those who are not directly affected by massive lay-offs or company bankruptcy (Sobotka, Skirbekk, and Philipov 2011; Hofmann, Kreyenfeld, and Uhlendorff 2017). The role of emotional factors in driving the economy is not new (*animal spirits*, Keynes 1936; Akerlof and Schiller 2010), but only recently has attention been given to the role of perceptions of economic conditions in family demography. Michaela Kreyenfeld (2010) pioneered a new generation of studies in which actual economic conditions are considered *vis-à-vis* perceptions (e.g., Kreyenfeld 2015; Bhaumik and Nugent 2011; Fahlén and Oláh 2018). She emphasized that people with the same employment conditions might differ in the way they operate fertility choices – because they feel, tolerate, and react to uncertainty in different ways or because of unobserved job-related amenities (Vignoli, Mencarini, and Alderotti 2020). Notwithstanding their importance, these perceptions, being contingent on the economic conditions of individuals, are still part of the “shadow of the past”. Economic uncertainty is – by its very definition – a forward-looking notion: it thus necessitates a framework that acknowledges its prospective nature.

## Fertility under conditions of uncertainty: The Narrative Framework

### Theoretical premises

To understand European fertility patterns and trends it is necessary to adopt a novel framework, one which takes into account that, under uncertain conditions, people can use their imaginative capacity to place themselves in one or more possible futures that cannot be deduced from the present. In a situation of uncertainty, past experiences and expectations come into play in an imaginative “dialogue” over the future, considering “competing possible lines of action”, because “deliberation is an experiment in finding out what the various lines of possible action are really like” (Dewey 1930 [1922]: 190).

Following the New Home Economics (Becker 1964, 1993), fertility decision-making is seen as a rational evaluation of the future expected utility of having children, with people calculating the trade-off between paid work and child-bearing. Empirical evidence suggests this substitution effect among women (Matysiak and Vignoli 2008, 2013). The model, however, assumes an unrealistic form of individual agency (*Homo oeconomicus*) in which partners *calculate* and *discount* the future costs and benefits of a child. Other explanatory models of demographic behavior apply psychological frameworks, as in the case of the Theory of Planned Behavior (TPB) (Ajzen 1991; Ajzen and Klobas 2013) and the Traits-Desires-Intentions-Behavior approach (TDIB) (Miller 1994, 2011). Both these explanatory models are mostly based on subjective perceptions of the personal condition and social norms. But they devote scarce attention to the future orientation of cognitive processes and their imaginative capacity. The simple substitution of socio-structural factors, with their subjective interpretation, may remain within a deterministic approach to explaining social actions, able to account for the influence of past and present conditions (mostly socialization, social norms and psychological traits). In other terms, they do not account for the human capacity to deviate from an expected course of action.

Recent advances in economic sociology maintain that economic decisions for long-term investments are taken under conditions of fundamental uncertainty over the future, and that imaginaries of the future have a central role in decision-making processes (Beckert 2016; Beckert and Bronk 2018). Investors, indeed, cannot forecast whether their investments will be successful or not: given that the long-term future is open to fundamental uncertainty, many elements may interfere with expected returns. Even if many microeconomic models assume that investors are mostly rational evaluators of alternatives, the empirical observation of the market functioning suggests that fictional expectations and imagined futures play a central role in the decision-making process within the economy. More generally, the whole capitalist dynamic of investment, consumption and profit is based on the entrepreneurs’ capacity to bring creativity and innovation into the markets (Keynes 1936). Innovation cannot be forecast. It is a real source of uncertainty for the economic dynamic, and fictional expectations and imagined futures are the fuel for this dynamic (Beckert 2016).

These developments in assessing the role of the future in the economic decision-making process provide us with useful insights for family demography research. Life-course decisions like fertility are always a first step into an unknown future that can never be comprehensively forecast. Expectations and imaginaries may play a crucial role in the fertility decision-making process, but they have not yet been considered in a systematic manner in demographic research. Following Beckert’s approach, we suggest that demographic behavior and fertility decisions are not the mere results of “*the statistical shadow of the past*” (Davidson 2010: 17), but they also depend on the “*shadow of the future*” (Bernardi, Huinink, and Settersten 2019: 4). Future expectations and imaginaries guide the decision-making process and produce real effects, irrespective of their level of truthfulness, rationality or plausibility.

### An outline of the Narrative Framework

Drawing on these contributions, we elaborate the Narrative Framework and argue that expectations, imaginaries and narratives of the future determine fertility decisions together with structural constraints and past experiences (Vignoli et al. 2020). When childbearing is planned, typical elements taken into account in the decision process are *structural constraints* such as, to give some examples, the level of education attained, employment, partnership and mobility experiences. These experiences are partly shaped by personal predispositions, like risk aversion or other personality traits, that may also exert a direct influence on fertility choices. Past experiences over the life course and present status are the standard elements employed as determinants of fertility intentions and behavior. Structural constraints alone cannot, however, predict reproductive behavior. Past and current personal circumstances, and their perceptions, synthesize the state-of-the-art for the vast majority of demographic studies. We can consider the sum of these elements as the *shadow of the past* influencing the decision-making process.

But fertility decisions are not merely a shadow of the past: expectations, imaginaries and narratives of the future also play a role. For example, uncertain labor conditions may not be seen as an obstacle to having a child in the light of strongly expected economic growth; or, they may inhibit fertility in the light of expected economic decline. *Expectations* are beliefs regarding future states of the world, anchored in the past and actual conditions. In addition, human agency has the capacity to influence the expected future or to deviate from an expected course of action. A wishful future of numerous descendants or a strong belief in the sacredness of family, for example, may encourage childbearing even in a condition of low household income with accompanying adverse economic expectations. The use of such *imaginaries* stems from the human capacity to place oneself in an imagined situation that cannot be deduced from present conditions. Imaginaries represent a powerful source of action: people can imagine a wishful future and, subsequently, try to reach this imagined future (Bronk 2009). The transition to the first child, in particular, is encouraged by positive imaginaries related to family and parenthood, partly shaped during childhood and adolescence. Instead, considering that in the case of the transition to additional children individuals already experienced parenthood, we can surmise that this experience re-shapes individuals’ imaginaries, or moderates their influence on the desire for a new child.

When imaginaries of the future are associated with a hypothetical course of actions and their causal interconnection, they constitute a (personal) *narrative of the future*. Structural constraints, (economic) expectations and imaginaries contribute to the definition of a narrative of the future driving the fertility decision-making process forward, where fertility may be chosen *despite* uncertainty about the future or avoided *according to* conditions of uncertainty. A narrative of the future can be seen as a powerful *anti-uncertainty device* (Boyer 2018), because it offers individuals the possibility of taking a fertility decision notwithstanding the uncertainty they face. The socio-psychological Uncertainty Reduction framework from Friedman et al. (1994) is, for instance, a source of narratives according to which having children may serve as a strategy for reducing biographical uncertainty. This theory contends that uncertainty reduction is a universal immanent value driving the choices of all rational actors, and that “*having a child changes life from uncertain to relatively certain*” (Friedman et al. 1994: 383). From this perspective, women may respond to unfavorable employment prospects by choosing the “alternative career” of becoming a mother. However, if personal narratives directly reflect a condition of economic uncertainty, they can also have negative effects on fertility decisions. When making vital life course decisions, the young were found to be more likely to postpone partnership and parenthood commitments when facing growing economic and temporal uncertainty (Mills and Blossfeld 2013). Given a specific set of opportunities and constraints, personal narratives may support the decision to postpone childbearing or, indeed, to not have children at all. Of course, a family imaginary may revolve around the desire to remain voluntarily childless, so that structural constraints and expectations only play a marginal role in defining fertility intentions.

Personal narratives perform four main functions, providing reasons for action (Hedström 2005; Uebel 2012). First, irrespective of the extent to which they may be false or actions questionable, personal narratives of the future select the key elements of the story and avoid what is considered irrelevant for the events at stake, thus reducing world complexity (*selection function*). Second, narratives of the future support people in interpreting and recognizing the analogies of the new elements at stake with already experienced elements, and they, then, classify them into binary oppositions (Lévi–Strauss 1963) (e.g., stable/precarious, enough/not enough, short-term/long-term) or within more complex relationships (*interpretation function*). Third, narratives make a given environment more intelligible and actionable because they identify the causal path for reaching the goal. The different options for reaching the imaginary can thus be compared thanks to the *causal modelling function* of a given narrative of the future, something that provides links between planned actions and expected outcomes. Importantly, the more the decision to be taken has important long-term effects, the more a conscious narrative of the future is needed to help with selection, interpretation, and causal modeling. Fertility is clearly an important and long-term decision. Finally, narratives of the future provide the rational and emotional motivation to sustain the efforts of dealing with uncertainty in the future, and reinforce the long-term commitment required to reach a given goal (*action support function*, Tuckett and Nikolic 2017; Tuckett 2018).

The interlinkages between personal narratives and fertility are far from being formed in a social vacuum. In the Narrative Framework, together with the micro-level dimension (where personal narratives are formed), meso-level (i.e. social networks) and macro-level (i.e. media and institutional context) dimensions are also relevant. Personal narratives of the future are, in fact, often based on *shared narratives*, that is narratives of the future adopted by relevant others such as parents and peers, or conveyed by the media. Shared narratives can be seen as the *building blocks* of personal narratives, and may stem from cultural preferences transmitted by the influence of parents and peers (Bachrach 2014). Moreover, the diffusion of (social) media provides increasing opportunities for social interaction among peers, as well as unprecedented access to relevant others’ opinions and experiences, which may influence an individual’s fertility decision-making process.

In sum, in the Narrative Framework, we posit that fertility decisions under economic uncertainty are not only related to structural constraints, but also to personal narratives of the future. Personal narratives are socially-constructed as they are embedded in existing cultural and institutional frameworks, as well as being influenced by media narratives. We now discuss in more detail the role of intergenerational narratives and those of peers and the media.

### Building-blocks of personal narratives: Intergenerational and peers’ narratives

Much of the existing research focusing on how individuals’ experiences of employment uncertainty affect their childbearing plans has a limit: it does not consider that individuals may react very differently based on what they perceive as the *necessary preconditions* for starting a family and for having children. Following Easterlin’s hypothesis (1976), these perceptions are influenced by a comparison individuals make between the socio-economic well-being of the previous generation and their own levels. Easterlin’s original thesis relates to income and claims that “relative affluence” is more important than the absolute level of economic endowments for fertility. In other terms: income matters for fertility decisions only in relation to individuals’ aspirations concerning the minimum acceptable standard of living, ideas which are shaped during socialization. A young man who entered the labor market during the *Trente Glorieuses* was able to improve on the standard of living he had experienced during his childhood. However, increasing income volatility and inequality have made this more difficult for subsequent generations, where young men and women may decide to postpone fertility decisions if their economic resources are perceived to be scarce relative to their aspirations. Extending Easterlin’s line of reasoning to job characteristics other than income, it can be argued that if a generation experienced particularly favorable labor market conditions – e.g. a smooth and predictable school-to-work transition and stable and full-life employment – these conditions will represent the minimum acceptable standard for the children of that generation, notwithstanding increasing structural economic uncertainty. In other words, the peculiar life circumstances experienced by the *baby boomers* shape the family imaginaries of their children and, in turn, the labor market expectations of these children.

Social perceptions related to the importance of labor market stability and predictability for childbearing plans are, though, likely to differ substantially across institutional and cultural contexts. As noted above, in Anglo-Saxon countries, employment-protection legislation was much looser than in other European areas already by the mid-1980s (Esping-Andersen 1990). Then, in Conservative and especially Mediterranean European countries, partial and targeted labor market reform contributed to strong labor market segmentation (Polavieja 2003; Barbieri 2011; Cutuli and Guetto 2013). The Post-Fordist young facing globalized labor markets in the UK are, thus, not as likely to feel the same *good-old-time* nostalgia as their Mediterranean counterparts. In the UK, young people enter the labor market under the same conditions as incumbent workers. In Southern European countries, instead, the young find themselves working side-by-side with their older, more protected counterparts.

Beyond the institutionally-driven causes of cross-country heterogeneity in social perceptions, sociocultural factors, too, have to be taken into account. It has been argued, indeed, that family values are related to both the demand for and actual labor market regulations, so that countries with *strong family ties* (Reher 1998) in Mediterranean Europe attach, culturally-speaking, more importance to job protection (Esping-Andersen 1990; Alesina et al. 2015). In addition, in a strong family setting, children are more likely to feel parental pressures relating to their family decisions, because of the longer stay in the family of origin and the latest-late age for leaving home (Billari 2004). Southern European parents are thus in a stronger position to influence their children’s aspirations concerning a stable and predictable life-cycle. This is a situation that should not apply to other Western European countries, where the influence of peers may be more relevant (Di Giulio and Rosina 2007; Guetto et al. 2016).

Drawing on the Narrative Framework, Southern European parents may influence their children in giving relevance to some elements (e.g., labor market stability and security) and not to others (*selection function*). As such they may evaluate one type of work contract (e.g., temporary employment) or living arrangement (e.g., cohabiting in a rented house) as being unsuitable for having children (*interpretation function*). Through these influences, they might affect their children’s perception of the pre-conditions for starting a family (*causal modelling function*). Finally, residential proximity and/or frequent intergenerational contacts reinforce a narrative of the future, shared between parents and children, that contributes to the postponement of the transition to adulthood (*action support function*).

To sum up, personal narratives of the future are not simply the products of idiosyncratic preferences and the psychological characteristics of individuals planning their family life in a social vacuum. They are shaped by culturally- and institutionally-rooted collective expectations and imaginaries conveyed by parents. However, because of the increasing pervasiveness of internet and social media, parents and previous generations are less likely now to represent the (only) benchmarks as young people form their expectations and imaginaries.

### Building-blocks of personal narratives: Media-channeled narratives

During the Great Recession, references to economic uncertainty featured prominently in public discourse. The Great Recession was popularized and spectacularized by a tsunami of news that favored a simplified narrative of the crisis as the “evil” hanging over contemporary European societies (Cepernich 2012). This was a novelty compared to previous recessions. In the case of the Great Depression of the 1930s, when a rapid surge in unemployment was followed by a drastic drop in fertility (Kiser and Whelpton 1953), information about the economic situation was not as widespread as in a digital, globalized age. Today, the reference groups with which individuals compare themselves have expanded globally. Another key building block of personal narratives is thus embedded in the role of shared media-channeled narratives. In times of uncertainty, even individuals who have not lost their jobs are worried about layoffs, reduced work hours, and limited job mobility. For example, those who have not experienced foreclosure might be reasonably concerned about declining home values and the possibility of falling behind on mortgage payments. For most citizens, the media represent their major source of information regarding the economic sphere (Joris, d’Haenens, and Van Gorp 2014; Joris, Puustinen, and d’Haenens 2018). They “set the economic temperature” in Europe and create images of society. The Great Recession and the Euro crisis saw the media creating a pessimistic image of a stagnant, underperforming continent in the public sphere. In addition to this, the rise of xenophobic attitudes related to the ongoing refugee crisis, Euroscepticism (e.g., the long drawn-out Brexit process may cast a shadow over both the UK and the rest of Europe), and populism, all fueled by widespread media discourse (Engesser, Fawzi, and Larsson 2017), challenge the emergence of a public sphere promoting social, cultural and political integration. In other words, media coverage has contributed to creating a sense of uncertain futures among European citizens.

Essentially, the media perform the main functions of narratives: not only do the media select the topics they report on (*selection function*), they also define the way they cover and frame these topics with respect to angles, tone, and so forth (*interpretation function*). This might affect individuals’ perception of the phenomena that surround them, and their causal interconnectedness (*causal modelling function*). Finally, media users often join online communities that tend to reinforce their beliefs, acting as echo chambers (*action support function*).

In the realm of fertility research, we only located a few studies on the role of media. A paper estimated the impact that the entry of cable TV had had on subjective measures of female autonomy, school enrollment, and fertility in India (Jensen and Oster 2009). In a similar vein, La Ferrara, Chong, and Duryea (2012) looked at the effect of television on fertility in Brazil. They found that, after introducing time-varying controls and time-invariant area characteristics, the presence of the Globo channel depresses fertility: Globo is the main producer of soap operas which portray small families. In a recent review of the effects of recession on fertility, Sobotka, Skirbekk, and Philipov (2011) emphasized the role of apprehension regarding future negative economic events in shaping fertility. They suggested that individuals’ observations into the broader economic climate, including, crucially, media coverage, might increase uncertainty and negatively affect fertility. Schneider (2015) examined the effect of area-level economic conditions on state fertility in the years leading up to and including the Great Recession in the United States. He suggested that press coverage comes closer to explaining fertility decisions than do measures of unemployment and foreclosure.

These examples are suggestive, but there is, as yet, no study of this kind for Europe. The constant overflow of information coming from the media is likely to play an important role in shaping individuals’ narratives about their future economic prospects, particularly after the Great Recession. We would suggest that more research is needed in addressing the role of media-channeled narratives about economic uncertainty in the study of fertility dynamics.

## Personal narratives of the future: Prospects for research

### Research schemes

**Fig. 2:**
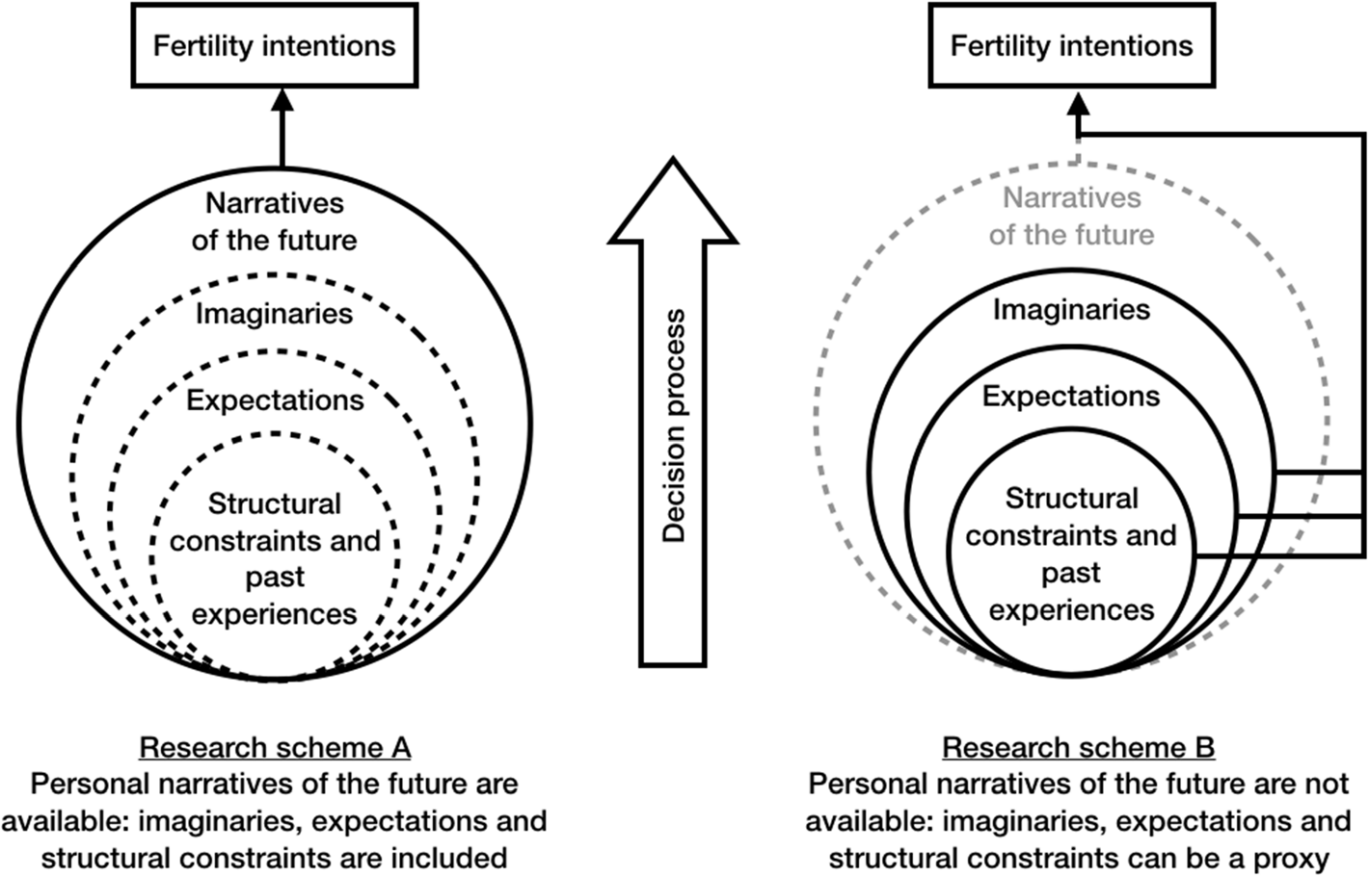
Research schemes in the Narrative Framework

Personal narratives of the future may be explored in various ways, depending on the information available. Whether personal narratives of the future can be directly investigated by the researcher, it should be possible to disentangle the role of all their constitutive elements (structural constraints, including shared narratives, expectations, and imaginaries) (Fig. 2, research scheme A). Most likely, this translates into qualitative investigations (“The study of narratives through qualitative investigation” section). Sometimes personal narratives are not accessible, however. If relevant information regarding structural constraints, expectations and/or imaginaries is known, it may be used as a proxy for personal narratives (Fig. 2, research scheme B). This translates into quantitative investigations (“The study of narratives in contemporary quantitative surveys” section). Experimental research might also be fruitful for operationalizing the role of narratives in fertility intentions research (“The study of narratives of the future with experiments” section).

Regarding shared narratives, if related measures are obtainable – e.g., through indicators of the media coverage of economic issues – their effects can be contrasted with those of objective indicators of economic uncertainty. The effects of shared media-channeled narratives on individuals’ fertility decisions can be interpreted in terms of their influence on the (unavailable) personal narratives of the future.

### The study of narratives through qualitative investigation

Qualitative analysis provides privileged methods for collecting personal narratives of the future that condense and synthesize the effects of structural constraints, expectations and imaginaries (Fig. 2, research scheme A). Semi-structured in-depth interviews represent a useful way of grasping the different functions performed by narratives in fertility decision-making (Randall and Koppenhaver 2004). Qualitative interviewing is a common method for the study of narratives in social sciences (Czarniawska 2004; Elliott 2005; Andrews, Squire, and Tamboukou 2008), but there are still not many examples of its use in studies of the fertility decision-making process in post-industrial (Western) countries (e.g., Bernardi, Klärner, and von der Lippe 2008; Mynarska 2010; Bernardi, Mynarska, and Rossier 2015; Bueno and Brinton 2019; Bueno 2019).

In the study of personal narratives and fertility intentions, the *selection* process can be assessed through questions that: directly focus on the major sources of concern that inhibit the fertility decision; or, alternatively, that indirectly explore sources of uncertainty and worry about the future. Then, after the identification of the key elements (*structural constraints*) that influence the fertility decision-making process, their meaning or degree of influence can be explored. The extent to which, for example, a lack of good housing options may influence a fertility decision depends on individuals’ subjective *interpretation*. During the analysis of an in-depth interview related to future family plans a researcher can explore the different sources of uncertainty by looking at the adjectives (e.g., enough/not enough, stable/precarious) attributed to the different elements of the story.

The narrative of the future provides and reflects the contingent plan for reaching personal imaginaries. Personal imaginaries represent an ideal future that can be investigated by asking people to describe their imagined long-term future in terms of partnership, family, housing, job, and other life course domains. Imaginaries often influence personal narratives of the future. For instance, in the case of people with a positive family imaginary and a strong desire to have children, but with still negative short-term fertility intentions, limitations (e.g., precarious job and housing conditions) are usually matched with plans to cope with said limitations (e.g., getting a full-time job will allow us to rent a better house that will allow us to try for a child). This *causal modeling* function of the narrative of the future can be explored with direct (e.g., what are the necessary aspects and conditions for you to decide to have a child?) or indirect questions (e.g., what do you think are the necessary aspects and conditions for a person to decide to have a child?). In an open stream of thought regarding future family plans within a non-scheduled interview, the causal path for reaching the imaginary can, instead, be identified *ex-post* during the analysis.

Family formation requires months or years of effort in reaching the imagined goal. In the daily routine or in facing unexpected negative events that may arise while going down this path, the family imaginary and the personal narrative of the future provide the necessary emotional resources and commitment. The *action support* function can be investigated with in-depth interviews, too. The interviewer can ask about “bad” moments during the family formation process. The imaginaries and narratives of the future that respondents mobilize in these moments show how narratives support motivation and a commitment to act.

Transitions to first and higher-order births need to be explored as unique life-changing events. Prior studies have emphasized that the transition to parenthood (that is, occurrence of first births) is especially affected by rising economic uncertainties (Blossfeld et al. 2006; Kreyenfeld et al. 2012). The transition to first child is mostly encouraged by positive imaginaries related to family and parenthood, often shaped during socialization, whereas the decision to have another child is also influenced by previous parenthood experience(s), whose memories generate expectations and may become an emotionally-charged support that encourages/discourages a new child. Fertility intentions (prospective) and fertility history (retrospective) are interwoven in the case of the transition to higher-order parities. They are distinct but interconnected processes that can be investigated in qualitative interviews. Fertility history, in particular, can be considered to be a special case of oral history (see Roberts 1995; Pagnini and Morgan 1996).

Qualitative methods also allow for a more open approach to narratives. Unstructured in-depth (or non-scheduled) interviews, in particular, allow the researcher to observe how people describe and frame their situation. It is possible to identify the relevant elements they consider in the fertility decision-making process, and their causal interconnection, in a neutral environment, without predetermined options and with virtually no bias on the side of the researcher (Glaser and Strauss 1967; Strauss and Corbin 1990). Unstructured in-depth interviews on fertility narratives may focus on the relevant moments that influenced the fertility decision. Important decisions, like fertility, are always connected to some specific moments when a complex combination of factors becomes an intelligible narrative of the future that allows for fertility decisions to be taken. The researcher can ask the respondent to remember a few salient moments that contributed to the final decision. These key moments can be evoked and narrated by the respondent in his/her own words, with related emotions and emphases (Tuckett 2011; Tuckett and Nikolic 2017). In this setting, people or factors influencing the fertility decision are not predetermined by the researcher, but they freely emerge in the personal narrative. The researcher encourages and sustains the dialogue without imposing a particular perspective, but takes care to reconstruct the personal narrative of the future in those specific key moments in which the fertility decision is made. During the analysis of the unstructured interviews collected, relevant factors contributing to the fertility decision will be identified, together with their personal interpretation (Strauss and Corbin 1990).

### The study of narratives through quantitative surveys

While personal narratives of the future and their functions can be more naturally studied through qualitative methods, the role of their constitutive elements – structural constraints including shared narratives, *expectations* and *imaginaries* – can be studied in quantitative research (Fig. 2, research scheme B). Clearly, a necessary starting point is the availability of information about *expectations* and *imaginaries*. Whereas few of the available surveys include economic expectations (see the paper’s Appendix), in conjunction with or in absence of information concerning fertility, the other constitutive elements of personal narratives of the future, that is imaginaries and shared narratives, are not usually available.

Surveys aimed at testing the role of narratives for fertility decisions should include questions on expectations about the future with regard to relevant domains, at the individual, family and contextual levels. At the individual level there should be, of course, objective indicators of individuals’ labor market and economic condition and the *current* subjective dimension of economic (in)security. But surveys should also include *forward-looking* measures of uncertainty – such as the perceived *stability* of the current job in the immediate future. Another important dimension is the concept of perceived *resilience* to adverse economic shocks, which could be operationalized through a question about the perceived chances of finding a new job with similar characteristics within a few months in case of job loss. Respondents might also be asked about their prospective financial uncertainty. Economic expectations should concern the perceived prospective situation of the partner and, more generally, of the (more or less close) context, too.

Measures of family imaginaries are even rarer in existing surveys, although common questions on the ideal number of children in one’s own life could represent a valuable proxy. Usually available questions on family values and adherence to social or religious norms, such as individuals’ belief in the “sacredness of family”, might prove relevant in defining personal family imaginaries. In order to include a forward-looking perspective, questions may ask how much having a(nother) child would make the respondent happy or how much the respondents consider a list of different aspects (e.g., family, children, job career, etc.) to be relevant for their futures. Of course, imaginaries can change during the life-course, and are likely to play a different role in the transition to parenthood and higher-order childbirths. For these reasons, the role of imagined futures could be grasped either retrospectively (e.g., “Since I was a child, I have always dreamed of becoming a mother/father”) or by offering the respondents a set of hypothetical futures (e.g., “At the age of 50, I see myself having at least two children”).

Exploring individuals’ family imaginaries through survey questions may be exposed to well-known biases due to social desirability – e.g. a childfree woman in a high-fertility society – and cognitive dissonance – e.g. questions concerning the importance attributed to children among women close to the end of their reproductive age who did not reach their imagined goals. Making use of the vignette technique might, here, represents a valuable alternative (Finch 1987; Schoenberg and Ravdal 2000; Hughes and Huby 2004). A series of vignettes could illustrate several types of families (married or cohabiting couples, with and without children) at various life-stages. Through specific close-ended questions, the researcher can analyze respondents’ reactions to the vignettes and bring their family imaginaries to light. Vignettes making use of pictures may be particularly useful in grasping imaginaries, which often refer to ideal-typical family settings (e.g., a couple with children sharing dinner in their home). The use of vignettes represents a potential innovation in family surveys that would clearly need to be validated in *ad hoc* pilot tests, and targeted analyses.

As regards the influence of media-channeled narratives, surveys should potentially include questions regarding the overall exposure to media (TV, internet, and social media) and the fruition of specific contents. Survey data could then be augmented with indicators of the media coverage of economic news. The question here is: does the media coverage of the economy affect fertility intentions and behaviors *net* of objective economic developments? Is the effect of shared narratives homogeneous or heterogeneous across societies or social groups?

In the Appendix, without claiming to be exhaustive, we go through some international surveys that include questions aimed at capturing different dimensions and nuances of uncertainty about future economic prospects. They all refer to expectations. This review suggests that the possibility of carrying out observational studies able to account for an operational distinction between structural constraints and perceived prospective uncertainty is clearly constrained by data availability. There are very few surveys collecting both forward-looking measures of uncertainty in the form of expectations and imaginaries coupled with fertility. Future data collection programs should make more efforts to include perceived prospective uncertainty together with fertility questions. At the time of writing, an *ad hoc* uncertainty module that includes specific forward-looking questions about uncertainty has been proposed for integration in the next round of the Generations and Gender Survey.

### The study of narratives through experiments

We conclude this section pointing to an alternative to the use of survey data for the quantitative analysis of personal narratives of the future: namely, the use of experiments. The social sciences are largely observational, characterized by the application of non-experimental methods such as surveys, interviews, or direct observation. Nevertheless, many social science questions may also be addressed using experimental methods, and some may be best approached in this way (Jackson and Cox 2013). This is especially true in the context of the research on decisions taken in conditions of uncertainty (Shmaya and Yariv 2016). In particular, questions aiming at assessing causal relationships are suitable for experimental investigation. In the experimental approach, the researcher manipulates real-world conditions, randomly assigns participants to those conditions, and observes the resulting outcomes. When used with participants who vary in theoretically relevant ways, experimental designs allow researchers to both investigate causal relations and to assess potential interactions between experimental conditions and the characteristics of the participant (Anderson and Mellor 2008; Singleton and Straits 2010). Importantly, in a laboratory experiment it is possible to account for individuals’ personal predispositions towards decision-making in a context of uncertainty. To this end, the use of lotteries is standard practice in experimental economics (Holt and Laury 2002; Anderson and Mellor 2008; Dohmen et al. 2011). Experiments might also be included in online or more traditional surveys, even if the researcher has a lower degree of control over the experimental premises and even if the use of lotteries is much less reliable.

Experiments in the social sciences have increased appreciably in recent years and even those disciplines that have traditionally eschewed experimental designs have witnessed an increase in experimental research (Dodoo, Horne, and Dodoo 2014). Experiments represent a common feature in behavioral economics, where the connection between narratives and individual and collective economic behavior is at the core of a specific line of research (Shiller 2019). The literature offers examples of experiments for assessing the causal impact of narratives on behavior in several fields such as marketing (Escalas 2007) or education (McQuiggan et al. 2008). In the realm of fertility research, to test the role of shared narratives of the future, mock newspaper stories and media clips could be used to manipulate the perception of a future situation, to provide more or less information, and to confront participants with different uncertain conditions (Starmer 2000) and frames (Tversky and Kahneman 1992). Societal crisis conditions can hardly be realistically simulated in the laboratory, but related perceptions and emotions can be. Previous experimental studies have managed to manipulate crisis concerns with visible effects on treated participants’ attitudes and intended behaviors (Druckman et al. 2011). Individuals’ fertility plans represent the outcome variable, which can be measured by asking participants whether they intend to have children in the (near) future. The use of online and laboratory experimentation represents an innovation for demographers and fertility intentions research, which have, to date, depended primarily on.

To the best of our knowledge, the use of experiments in family demography is currently explored only within the EU-FER project (www.eu-fer.com). One of the pillars of the project is the use of online and laboratory experiments to test the role of shared narratives of the future state of the economy in shaping fertility intentions. After reading mock stories about a future economic scenario, individuals’ fertility plans are measured by asking participants whether they intend to have children in the next three years. EU-FER experimental evidence aims to provide internal validity to causal claims about the impact of economic uncertainty on the fertility decision-making process. Laboratory experiments are organized in Italy and Norway, whereas online experiments are carried out in Italy, Norway, Germany, Poland, and the UK. These are countries with different family formation patterns, influenced by different cultural, political and economic circumstances. Cross-country experiments highlight similarities across societies and draw out country-specific distinctions on the impact of perceived economic uncertainty on fertility plans.

## Concluding remarks

“*Whatever it takes*”: these three simple words of the then President of the European Central Bank (ECB), Mario Draghi, proved to be enough to see off global financial speculation against the Euro in 2012. Of course, the ECB was (apparently) prepared to unleash its monetary power, but that turned out to be unnecessary: the narrative of a determined ECB sufficed. During the Coronavirus pandemic emergency in 2020, ongoing at the time of writing, the new President of the ECB Christine Lagarde provoked the biggest crash in the history of the Milan stock market by simply saying that the function of the ECB is not to “*close spreads*”. These examples show how actors take decisions on the basis not only of structural constraints, but also of shared and personal narratives of the future.

In this reflection, we have connected fertility research to novel research approaches in other disciplines (Beckert 2016; Beckert and Bronk 2018). We suggest that the focus of contemporary fertility studies should partly shift to assessing: how people build their narratives of the future to act according to or in spite of uncertainty; and, also, how these narratives are (not) related to objective economic constraints and their subjective perception. We do recognize, of course, that individuals differ in their ability to take family decisions under uncertain circumstances. This is based on their preferences and psychological characteristics, such as subjective well-being or risk aversion. Nonetheless, in a context in which (bounded) rational calculations of opportunities and constraints concerning fertility decisions are made difficult by increasing uncertainty, socially-constructed personal narratives of the future may become important frames in channeling individual action. Narratives help people to take decisions, reconciling the core contradictions of an uncertain future, so that they can find a conviction for long-term commitments including childbearing and parenthood. The building blocks of this kind of personal narratives are not idiosyncratic factors such as preferences or attitudes: they are deeply embedded in the cultural and social environment, as they are mediated through the shared narratives produced by agents of socialization, above all, parents, peers, and the media.

We believe that a focus on narratives of the future will help scholars to reach a better understanding of European fertility patterns and the reasons behind the current fertility winter in Europe. For instance, high labor instability seemed not to affect fertility trends in the UK, while fertility was affected by flexibilization reforms in Central and especially, Southern European countries. This may be explained, at least in part, by the different incidence of narratives that consider employment stability and life-course predictability as necessary preconditions for starting a family and having children. On the other hand, similar effects may be observed in contexts characterized by very different levels of *structural* economic uncertainty – in terms of general economic trends and unemployment levels. This was the case with the fertility drops in Nordic and Southern European countries following the Great Recession. Due to the prevalent narratives of the future, people react differently to the same sources of objective uncertainty (e.g., labor market insecurity in liberal or in coordinated economies) or certainty (e.g., the condition of full employment and inclusive welfare in Nordic countries before and after the Great Recession). More research is needed to address the role of personal and shared narratives in the study of fertility dynamics in an era of global uncertainty. The combination of quantitative results on shared (peers, parental, and media) narratives with the qualitative results on personal narratives might allow for a robust understanding of the influence of narratives of the future in the fertility decision-making process.

Three clarifications regarding the role of narratives are in order, here, as we conclude. First, socially-influenced personal narratives of the future do not necessarily foster fertility. Rather, they often suggest solutions to the uncertainty that induce a postponement in family transitions: for example, parental expectations concerning employment stability and life-course predictability as preconditions for fertility. A similar argument holds for narratives on economic uncertainty conveyed by the media. For instance, in the case of Nordic countries we may expect that, together with apparently positive objective indicators of social and economic context for fertility plans, the emergence of negative shared narratives of the future, related to a rise in global economic uncertainty, may explain the fertility drop.

Second, our arguments do not imply any clash between *structuralist* and *culturalist* explanations for fertility. Our aim is to suggest that the effects of objective economic situations on fertility might be moderated – exacerbated or attenuated – by personal and shared narratives. The systematic relationship between the objective and the subjective dimensions of economic uncertainty – in the form of personal and shared narratives – represents a crucial point to be addressed in future research. For instance, our argument on the role of previous generations and parental narratives does not imply a strictly culturalist explanation for the low fertility of Southern European countries in the last three decades. Rather, we see parental narratives as a complement, not a substitute, for explanations stressing the role of flexibilization *at the margins* and the sub-protective welfare system characteristic of Southern European countries (Barbieri 2011). In the same vein, the level of economic uncertainty narrated in the press and social media is likely to be correlated with underlying levels of unemployment and foreclosures in that region, but the media narrative is distinct in capturing online attention to those economic trends. It will be crucial to analyze whether public discourses on the crisis affect childbearing plans over and above the effects of more objective measures such as GDP or the unemployment rate. It will also be interesting to consider whether there are interaction effects between indicators of media-channeled uncertainty and aggregate economic measures.

Third, it is worth recalling that fertility analysis should be anchored in life-course research, which is concerned with the inter-linking of different life domains in structuring individual life courses. Fertility choices need to be conceptualized as a succession of transitions (or non-transitions) in one’s life-course (Kravdal 2002). This principle translates into the need to consider each parity progression as a separate phenomenon, recognizing that often paths into childlessness represent a distinct process (Mynarska et al. 2015; Miettinen et al. 2015). Addressing the economic uncertainty-fertility nexus from a life-course perspective also means recognizing that forms of family behavior are intertwined within individuals and over time: fertility does not occur in isolation, it emerges within relationships. Economic uncertainty may lead, not only to the postponement/avoidance of fertility, but to the postponement of marriage or to uncertain relationships (Vignoli, Tocchioni, and Salvini 2016). As a corollary, then, fertility careers need to be examined side by side with relationship and partnership careers.

## Data Availability

No dataset was used to write this paper.

## Appendix

From 1994 to 2002, the University of Wisconsin collected data on a sample of American households to examine how Americans perceived their short-term futures in relation to income and jobs (Survey of Economic Expectations, SEE). This survey cannot help current researchers to explore whether and how prospective economic uncertainty affects fertility (no measures of childbearing were included in the survey), but it proved its efficacy in testing novel job-related and financial uncertainty measures. It showed, indeed, how individuals in various demographic, social and economic circumstances have considerably different perceptions of uncertainty (Dominitz and Manski 1997; Manski and Straub 2000). Another study that is worth acknowledging here is the EU-funded research project Psycones (Psychological Contracting across Employment Situations; De Cuyper and Isaksson 2017), which includes several indicators of job, employability and contract expectations looking at the immediate future. These items have been used to study the impact of job insecurity on health and well-being (Höge et al. 2020): there is unfortunately a lack of questions about fertility (intentions). Finally, Eurobarometer – the multi-topic, pan-European surveys undertaken for the European Commission since 1970 – is also worth flagging up. Since the 1990s, the survey investigates the expectations for the future in terms of economic, financial and job situations at the individual level. Moreover, to the best of our knowledge this is the only survey asking for future economic expectations at the national, EU and world level. Again, no measures of fertility are included. SEE, Psycones, and Eurobarometer are nonetheless of primary importance for understanding the relevance of uncertainty – especially its perceptions – in the lives of individuals. They provide us with potentially useful questions for future survey planning.

Moving to social surveys including information about fertility, we can rely on both longitudinal (panel and/or retrospective) and cross-sectional surveys. If the data are longitudinal, uncertainty measures can be used to study fertility behavior; if the data are cross-sectional, they can be used as predictors of fertility intentions. Among the longitudinal surveys most frequently employed in family and fertility research, there are the German Socio-Economic Panel (G-SOEP), and the British Household Panel Survey (Understanding Society, USoc). Besides collecting *traditional* objective measures of uncertainty (e.g., unemployment episodes), they also ask whether the respondents are worried about their employment or financial situation. Although the measures present in G-SOEP look at the *future* in a vague way, they have been used in empirical analyses as proxies of individual perception of future economic uncertainty (Kreyenfeld 2010, 2015; Bhaumik and Nugent 2011); USoc data look, on the other hand, at one-year prospects, and though some recent works addressed the role of uncertainty in individuals’ lives (Berrington 2020), the link with fertility is still under-investigated. Job prospects are also included in the Survey on Household Income and Wealth – SHIW, a biannual panel survey carried out in Italy by the Bank of Italy – and in the Swiss Household Panel (SHP). The SHP has been exploited by a number of scholars, including Hanappi et al. (2017) who explored the relationships between changes in employment uncertainty, fertility intentions and their subsequent realizations. HILDA, the survey on Household, Income and Labour Dynamics in Australia, adds, as well, a measure of employability (i.e. resilience) to questions on job uncertainty. In addition to fertility intentions, the Generations and Gender Survey program (https://www.ggp-i.org) has very recently incorporated, in the last version of its questionnaire, one question about the perceived likelihood of losing one’s job (for both partners), and a question about the perception of one’s future income.

In cross-sectional surveys, too, forward-looking measures of uncertainty – coupled with fertility intentions questions – are rare. In the European Social Survey (ESS), for instance, only subjective perceptions of current personal circumstances are available. Questions that might allow researchers to grasp expectations of economic uncertainty can be derived from Trustlab, a project launched by OECD in 2016 in several countries (https://www.oecd.org/sdd/trustlab.htm). This project combines, in the Italian version, information on fertility plans in the immediate future and questions about both economic stability and resilience perception in the next year (Aassve et al. 2018).

**Table:**

Survey	Measure	Question formulation	Fertility
SEE—Survey of Economic Expectations	Employment uncertainty	– What do you think is the percent chance that you will lose your job during the next 12 months? – What do you think is the percent chance (or chances out of 100) that the job you eventually find and accept would be at least as good as your current job, in terms of wages and benefits?	No
	Financial uncertainty	– What do you think is the percent chance (or the chances out of 100) that your total household income, before taxes, will be less than Y over the next 12 months?	
Psycones	Employment uncertainty	– Chances are, I will soon lose my job. – I think I might lose my job in the near future. – I am sure I can keep my job. (For each item: strongly disagree; somewhat disagree; partly agree, party disagree; somewhat agree; strongly agree)	No
	Employability (resilience perspective)	– I am confident that I could quickly get a similar job. – I will easily find another job, if I lose this job. – I am optimistic that I will find another job, if I look for one. – I can easily switch to another employer, if I wanted to. (For each item: strongly disagree; somewhat disagree; partly agree, party disagree; somewhat agree; strongly agree)	
	Contract expectations	Only to be asked to non-permanent employees – I think I will be employed in this organization for longer than has been agreed in my employment contract. – I expect that I will have to leave here once my present employment contract has run out. – I think my present employment contract will be renewed when it expires. – I have been promised that I will get a permanent contract when my present contract expires. (For each item: strongly disagree; somewhat disagree; partly agree, party disagree; somewhat agree; strongly agree)	
EURO—BAROMETER	Future expectations	What are your expectations for the next 12 months: will the next 12 months be better, worse, or the same, when it comes to…? – Your life in general – The economic situation in (your country) – The financial situation of your household – The employment situation in (your country) – Your personal job situation – The economic situation in the EU – The economic situation in the world	No
G-SOEP—German Socio-Economic Panel	Employment uncertainty	For employed individuals: – How confident are you about your job security? (very concerned, somewhat concerned, and not concerned at all) For unemployed individuals: – How confident are you about finding a new job? (easy, difficult, and almost impossible)	Longitudinal study
	Financial uncertainty	– How confident are you about the household’s financial prospects? (very concerned, somewhat concerned, and not concerned at all)	
USoc—Understanding Society	Employment uncertainty	– I would like you to think about your employment prospects over the next 12 months. Thinking about losing your job by being sacked, laid off, made redundant, or not having your contract renewed, how likely do you think it is that you will lose your job during the next 12 months? Is it... very likely; likely; unlikely; very unlikely?	Longitudinal study (including intentions)
	Financial uncertainty	– Looking ahead, how do you think you will be financially a year from now, will you be... better off; worse off than now; about the same?	
SHP—Swiss Household Panel	Employment uncertainty	– Would you say that your job is very secure, quite secure, a bit insecure, or very insecure? – How do you evaluate the risk of becoming unemployed in the next 12 months?	Longitudinal study (including intentions)
SHIW—Italian Survey on Household Income and Wealth	Employment uncertainty	– How likely is it, according to you, that you will keep that job for the next 12 months? (on a 0-100 scale)	Longitudinal study (including intentions)
HILDA—Household, Income and Labour Dynamics in Australia	Employment uncertainty	For employed individuals: – I would like you to think about your employment prospects over the next 12 months. What do you think is the percent chance that you will lose your job during the next 12 months? (By loss of job, I mean getting fired, being laid off or retrenched, being made redundant, or having your contract not renewed). For not employed individuals: – I would like you to think about your employment prospects over the next 12 months. What do you think is the percent chance you will find a suitable job during the next 12 months?	Longitudinal study (fertility history)
	Employability (resilience perspective)	– If you were to lose your job during the next 12 months, what is the percent chance that the job you eventually find and accept would be at least as good as your current job, in terms of wages and benefits?	
GGS—Generations and Gender survey	Employment uncertainty	– How likely is that you will lose your job in the next 12 months? – How likely is that your partner will lose his/her job in the next 12 months?	Longitudinal study (including intentions)
	Financial uncertainty	– Do you think that your financial situation will get better or worse or will be about the same in 3 years from now?	
Trustlab	Employment uncertainty	– How likely do you think it is that you will still have a job in 6 months (if you have one now)? (from very unlikely to very likely, on a 0–10 scale)	Fertility intentions (Italy only)
	Employability (resilience perspective)	– If you were to lose your job, how likely is it that you would find a job with a similar salary within 6 months? (from very unlikely to very likely, on a 0–10 scale)	
	Financial uncertainty	– When it comes to the financial situation of your household, what are your expectations for the 12 months to come, will the next 12 months be better, worse, or the same?	
ESS—European Social Survey	Present job security	– My job is secure (not at all true, a little true, quite true, very true).	Fertility intentions
	Financial security	– Which of the following descriptions comes closest to how you feel about your household’s income nowadays? (finding it very difficult; finding it difficult; coping on present income; living comfortably)	
